# P-2213. Lung Microbiome Diversity Associated with Hospital Mortality in Intubated Patients Admitted to the ICU

**DOI:** 10.1093/ofid/ofae631.2367

**Published:** 2025-01-29

**Authors:** Ingrid G G Bustos, Jennifer M Baker, Christopher Brown, Nicole Falkowski, Piyush Ranjan, Lina Mendez, Natalia Sanabria-Herrera, Robert Dickson, Luis Felipe F Reyes

**Affiliations:** Universidad de La Sabana, Chía, Cundinamarca, Colombia; Michigan University, Ann Arbor, Michigan; Michigan University, Ann Arbor, Michigan; Michigan University, Ann Arbor, Michigan; Michigan University, Ann Arbor, Michigan; Clinica Universidad de La Sabana, Chía, Cundinamarca, Colombia; Clínica Universidad de La Sabana, Chía, Colombia, Bogota, Distrito Capital de Bogota, Colombia; University of Michigan, Ann Arbor, MI; Universidad de La Sabana, Chía, Cundinamarca, Colombia

## Abstract

**Background:**

The lung microbiota of healthy individuals tends to remain stable, but dysbiosis disrupts pulmonary homeostasis, impacting respiratory illness severity and mortality rates. Understanding lung microbiome alterations and their impact on clinical outcomes in ICU-admitted patients has been proposed as a crucial point to improve clinical outcomes, yet this area still needs to be explored. Therefore, this study aims to investigate microbial composition in the lower respiratory tract and assess whether microbial diversity in mechanically ventilated ICU patients is linked to mortality.**Figure 1.** Beta Diversity Comparisons in the Respiratory Microbiomes of BAL Samples from ICU Admitted Patients Stratified by Hospital Mortality.

**Figure:**
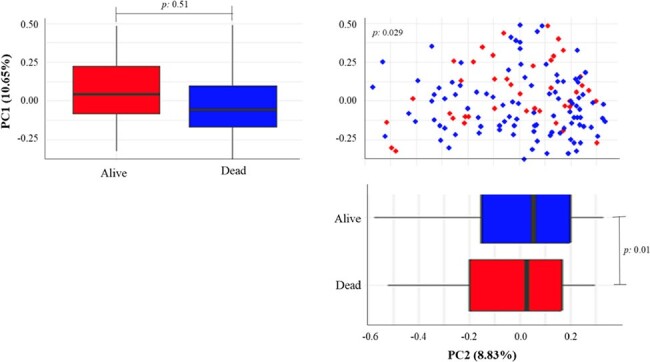
The beta diversity of bronchoalveolar lavage (BAL) samples from ICU-admitted patients is illustrated through a PCoA scatter plot utilizing Bray-Curtis distances, showing a significant albeit modest differentiation between groups (p = 0.029). The variance explained by each principal coordinate is delineated in the axis titles (p = 0.51 for PC1 and p = 0.01 for PC2).

**Methods:**

Conducted at Clinica Universidad de La Sabana in Colombia from January 2020 to July 2022, this prospective cohort study enrolled ventilated patients within 12 hours of ICU admission. Bronchoalveolar lavage samples were collected on the day of intubation. The operational taxonomic units (OTUs) were identified using 16S ribosomal RNA (rRNA) gene sequencing. Statistical analyses were performed using RStudio.**Figure 2.** Comparison of Relative Abundance of OTUs Stratified by Hospital Mortality in ICU Admitted Patients.

**Figure:**
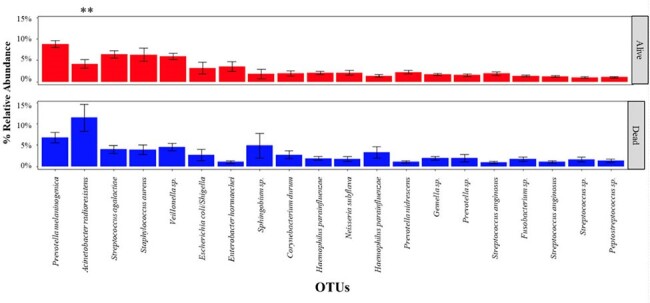
Percent of relative abundance of the top 20 most abundant microbes in the lungs. Student's t-test was used to calculate the p-value. Asterisks denote the level of significance ​observed: * = p ≤ 0.05; ** = p ≤ 0.01; *** = p ≤ 0.001.​

**Results:**

The analysis of 141 intubated patients revealed a predominantly male population (65% [91/139]) with a median (IQR) age of 50.0 years (30.5-67.0). Sequencing generated 20808915 reads and identified 18477 OTUs, with an average of 130873 reads and 13 OTUs per bronchoalveolar lavage sample. Subsequent refinement significantly reduced OTUs to 2096. Comparative analysis, employing Bray-Curtis distances, indicated a statistically significant differentiation (p = 0.02) between patients who died during hospitalization and survivors (Fig. 1). The primary components explained 73.2% of the data variance (R^2=0.73). Prevotella, Acinetobacter, Streptococcus, Staphylococcus, and Veillonella were the predominant OTUs identified in terms of relative abundance, although no significant differences were observed between groups (Fig. 2).

**Conclusion:**

Dysbiosis of the lung microbiome, characterized by a reduction in the beta diversity and increased concentration of Acinetobacter, was associated with increased mortality in patients requiring invasive mechanical ventilation at ICU admission. These findings highlight the importance of comprehending lung microbial alterations in ICU settings and their potential implications for intubated patients.

**Disclosures:**

All Authors: No reported disclosures

